# Hybrid FES-exoskeleton control: Using MPC to distribute actuation for elbow and wrist movements

**DOI:** 10.3389/fnbot.2023.1127783

**Published:** 2023-04-06

**Authors:** Nathan Dunkelberger, Jeffrey Berning, Eric M. Schearer, Marcia K. O'Malley

**Affiliations:** ^1^Department of Mechanical Engineering, Mechatronics and Haptics Interfaces Laboratory, Rice University, Houston, TX, United States; ^2^Center for Human Machine Systems, Department of Mechanical Engineering, Cleveland State University, Cleveland, OH, United States

**Keywords:** model predictive control (MPC), hybrid control (HC), functional electrical stimulation (FES), movement assistance, upper limb exoskeleton

## Abstract

**Introduction:**

Individuals who have suffered a cervical spinal cord injury prioritize the recovery of upper limb function for completing activities of daily living. Hybrid FES-exoskeleton systems have the potential to assist this population by providing a portable, powered, and wearable device; however, realization of this combination of technologies has been challenging. In particular, it has been difficult to show generalizability across motions, and to define optimal distribution of actuation, given the complex nature of the combined dynamic system.

**Methods:**

In this paper, we present a hybrid controller using a model predictive control (MPC) formulation that combines the actuation of both an exoskeleton and an FES system. The MPC cost function is designed to distribute actuation on a single degree of freedom to favor FES control effort, reducing exoskeleton power consumption, while ensuring smooth movements along different trajectories. Our controller was tested with nine able-bodied participants using FES surface stimulation paired with an upper limb powered exoskeleton. The hybrid controller was compared to an exoskeleton alone controller, and we measured trajectory error and torque while moving the participant through two elbow flexion/extension trajectories, and separately through two wrist flexion/extension trajectories.

**Results:**

The MPC-based hybrid controller showed a reduction in sum of squared torques by an average of 48.7 and 57.9% on the elbow flexion/extension and wrist flexion/extension joints respectively, with only small differences in tracking accuracy compared to the exoskeleton alone.

**Discussion:**

To realize practical implementation of hybrid FES-exoskeleton systems, the control strategy requires translation to multi-DOF movements, achieving more consistent improvement across participants, and balancing control to more fully leverage the muscles' capabilities.

## 1. Introduction

There are ~291,000 people in the United States living with spinal cord injuries, and the majority of these are cervical level injuries, resulting in tetraplegia (NSCISC, 2019). Injuries at such a high level of the spinal cord create severe arm and hand disabilities, resulting in an inability to complete Activities of Daily Living (ADLs). As a result, 71% of individuals with tetraplegia currently require assistance with ADLs (Collinger et al., 2013). Given this, it is not surprising that restoration of arm and hand function is a top priority among people with tetraplegia due to cervical spinal cord injuries (SCI) (Anderson, 2004). With scarce rehabilitation and assistive technology options, these individuals are largely dependent on full-time caregivers for feeding, grooming, and many other activities of daily living. Regaining the ability to perform these tasks independently will reduce requirements on caregivers and increase opportunities for individuals to return to social participation in their communities, both of which are highly correlated to quality of life (Dijkers, 1997).

Recovery of arm and hand function through rehabilitation can be achieved for individuals with some residual muscle capability (Dietz et al., 2002; Beekhuizen and Field-Fote, 2005), and there are promising results that show that the same intensive robotic rehabilitation that has been successful for inducing plasticity and recovery following stroke (Reinkensmeyer et al., 2000; Charles et al., 2005; Lum et al., 2012; Blank et al., 2014) can be effective for SCI (Kadivar et al., 2012; Fitle et al., 2015; Francisco et al., 2017; Frullo et al., 2017; Yozbatiran and Francisco, 2019). For those *without* residual motor capability, however, or for those for whom rehabilitation interventions have not been able to restore functional movement, assistive technologies are a more viable option for replacing lost function. Such approaches incorporate mechanical devices that are attached to the limb and have the capability to move the limb or hand, or approaches that electrically stimulate the existing muscles, causing muscle contraction and inducing motion of the upper limb.

Functional electrical stimulation (FES) is a promising assistive technology to restore arm and hand function. By activating a person's own paralyzed muscles via surface electrodes placed on the skin or surgically implanted electrodes, limb movements can be generated. This approach requires very low energy consumption and exhibits high embodiment by the person; however, FES cannot produce sufficient torques to enable whole-arm reaching movements in people with tetraplegia, as many muscles are unresponsive to FES (Peckham et al., 1976; Mulcahey et al., 1999). Further, general multi-joint motions are notoriously hard to control with FES even with the most advanced systems (Ajiboye et al., 2017), often resulting in fine-tuned feed-forward implementations due to the physiological delays in muscle response to applied stimulation, and difficulty in accurately modeling the response to muscle activation. Augmenting FES with an assistive robot offers additional torque to support whole arm reaching while also offering improved movement accuracy, but this comes at the expense of increased bulkiness and decreased wearability of the combined FES-robotic system. An optimal combination of FES and an assistive robot would maximize the contribution of FES to minimize size and power requirements of the robot (Dunkelberger et al., 2020).

**Figure 1:**
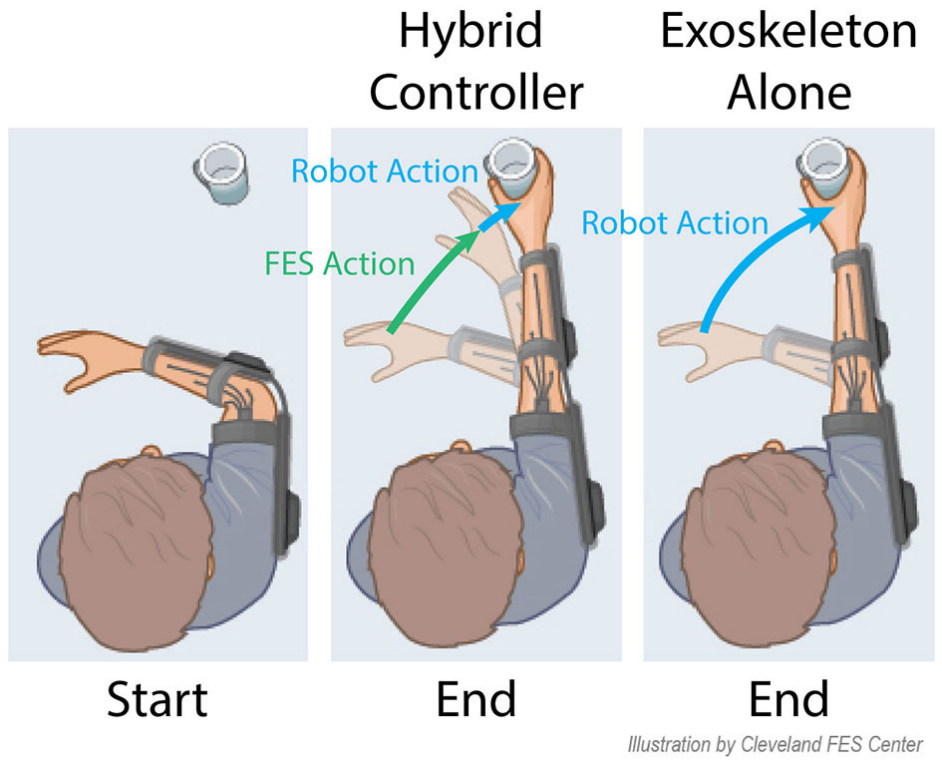
An example future application of hybrid systems is shown for a reach and grasp task. The incorporation of both FES and a robot allows for a large portion of the movement to be provided by FES, and the robot can provide small amounts of power to provide minimal movement corrections. With the robot alone, all power for the movement must be provided by the robot.

This combination of FES with robotic devices is starting to gain traction, and is termed hybrid FES-robot (or FES-exoskeleton) control. A conceptual representation of using FES with a robot is shown in Figure 1, where both robotic and FES action can complement each other to assist in the completion of activities of daily living. Many of the early approaches to bring this concept to reality did not truly combine and coordinate the actuation strategies for upper limb movements (Dunkelberger et al., 2020). Instead, each of the actuation types was used to achieve separate functions. For example, robotic devices have been used to lock degrees of freedom (Klauer et al., 2014; Ambrosini et al., 2017) or as gravity compensation (Cannella et al., 2016) enabling the muscles to relax and preventing fatigue. Other works have used robotic support devices to actuate one set of degrees of freedom, while FES is used to actuate another set (Varoto et al., 2008; Schulz et al., 2011; Ajiboye et al., 2017). Typically the robot controls motions that need precision or require larger torques and forces to support, such as elbow flexion and extension, while FES is used for coarse movements, such as grasping. For upper limb motions with coupled degrees of freedom, such as shoulder, elbow, and wrist movements, these existing control strategies pit FES against a robot-imposed locked-joint, gravity, or single-joint motion constraint, essentially wasting the free actuation from FES and transferring it to the robot. Recently, single-joint hybrid systems that do share actuation on the same joint have been explored, but research has been limited, testing only in the elbow flexion extension joint with biceps electrodes in a minimum jerk trajectory (Wolf et al., 2017; Burchielli et al., 2022), or in simulation (Bardi et al., 2021).

In lower limb applications, more advanced hybrid control algorithms have been explored, largely enabled by the repetitive nature of gait motions (Bulea et al., 2014; del Ama et al., 2014; Ha et al., 2016). These lower limb hybrid systems often use a version of iterative learning control that takes advantage of the repetitive movements to fine-tune control over several cycles. Some recent research has begun to use model predictive control (MPC) algorithms, which can be more readily adapted to non-cyclic movements in the lower limbs (Kirsch et al., 2018; Bao et al., 2021), and which are more similar to the non-cyclic movements required of upper-limb movements. Results from these studies using MPC have shown the ability to follow a step reference trajectory and hold a position, and the algorithms should generalize to arbitrary trajectories.

A truly shared approach for hybrid FES and robotic control of upper limb reaching movements is needed to combine these techniques in a manner that achieves generalized upper limb movement assistance in an optimal manner. In this paper, we present a model-based control approach to hybrid FES-exoskeleton control. Recent works have demonstrated the first steps toward this vision. Wolf and Schearer (2022) demonstrated the use of model-based algorithms to power FES in combination with gravity compensation from a robot. Our group has also demonstrated shared control of elbow flexion and extension movements with FES and exoskeleton assistance acting in coordination to follow a desired trajectory (Dunkelberger et al., 2022b). In that work, we showed that a model-based controller for our upper limb exoskeleton, which has knowledge of the expected contributions of FES, requires significantly less robot torque than a standard PD control algorithm, with minimal loss in trajectory following accuracy. Here, we expand our initial demonstration along a number of fronts. First, we present an MPC algorithm that removes the integral term used previously and incorporates an additional proportional-integral-derivative (PID) controller acting in parallel, resulting in improved performance in both trajectory following and reduction in torque requirements from the exoskeleton compared to our initial controller. We incorporate a sophisticated model of the user's arm dynamics that accurately captures behavior across the exoskeleton workspace. We experimentally demonstrate the performance of the hybrid FES-exoskeleton controller in able-bodied participants completing two trajectories for two degrees-of-freedom of the exoskeleton (elbow flexion-extension and wrist flexion-extension), and we compare the performance of the hybrid controller to an exoskeleton-alone case, as illustrated in Figure 1. Finally, we examine longitudinal performance of the hybrid FES-exoskeleton control for a subset of participants to determine how performance changes 1 week after the initial experiment trials.

## 2. Materials and methods

### 2.1. Participants

Nine able-bodied participants (four female, avg age 22.9) participated in a single session of the experiment after providing informed consent. Three of the nine participants, who had experience with FES prior to the initial experimental session, also completed a second session of testing using the same protocol at least 1 week after their first experimental session. The study was approved by the institutional review boards at Rice University (IRB #FY2017-461) and Cleveland State University (IRB #30213-SCH-HS).

### 2.2. Procedure

The goal of this study is to develop a new hybrid controller that distributes actuation between an exoskeleton system and an FES system. The goal of such a controller is that it can reduce the power requirements in comparison to an exoskeleton alone system, which can lead to more portable devices in the future that can assist individuals with SCI in completing general activities of daily living. To test the effectiveness, the developed hybrid controller is used to provide movements on two different degrees of freedom (DOF), elbow flexion/extension, and wrist flexion/extension. To understand how this compares to available exoskeleton systems, the resulting torque and position profiles for the hybrid controller are compared with an exoskeleton-alone controller in following two different trajectories.

### 2.3. Materials

The hybrid FES-exoskeleton system is comprised of two main subsystems that provide actuation. The first subsystem, which provides FES, is a transdermic electrical stimulation system (Trier et al., 2001) which provides eight output channels of bipolar stimulation. In this study, two channels are used for the elbow flexion/extension joint, and two channels are used for the wrist flexion/extension joint. To provide varying levels of output using the FES subsystem, the amplitude and frequencies are kept at a constant value for each channel, and the pulsewidth is varied.

The second subsystem is the robot, the MAHI Open Exoskeleton (Dunkelberger et al., 2022a). This robot provides four DOFs of movement support, namely elbow flexion/extension, forearm pronation/supination, wrist flexion/extension, and wrist radial/ulnar deviation, and each of these joints line up with the equivalent anatomical degree of freedom of a person using the exoskeleton. These will also be referred to by joint number throughout this paper, which are joints 1–4, respectively. The exoskeleton has an adjustable counterweight to account for varying arm masses, an adjustable slider to account for varying forearm lengths, and an adjustable shoulder abduction angle to keep the participant comfortable. The counterweight and forearm slider parameters are adjusted for each subject at the beginning of the experiment, and locked for the experiment duration. This shoulder abduction angle was kept at a value of 30° for all participants.

### 2.4. Methods

The study consists of several model characterization steps related to each of the subsystems, followed by experimental testing of the hybrid controller which makes use of these characterizations. First, the electrodes are placed in appropriate locations, and comfortable ranges of stimulation are found. Recruitment curves are characterized for each set of electrodes to define the relationship between commanded pulse width and muscle activation level. Gaussian process regression models are created to characterize torque output for each electrode based on the orientation of the upper-limb. The mass properties of the participant's arm are then characterized so that a combined dynamic model can be created for the arm-exoskeleton subsystem. The hybrid controller is created using the characterizations of each of the components. These characterization steps are more completely described in Sections 2.4.1–2.4.5. The hybrid controller is then compared against an exoskeleton alone controller in a scenario of following two trajectories for each DOF.

In this study, the elbow flexion/extension and wrist flexion/extension DOFs are tested independently. Each of the experimental steps is performed with the elbow flexion/extension joint and corresponding electrodes, followed by the wrist flexion/extension joint with corresponding electrodes. The explanations that follow apply to both DOFs.

#### 2.4.1. FES electrode placement

**Figure 2:**
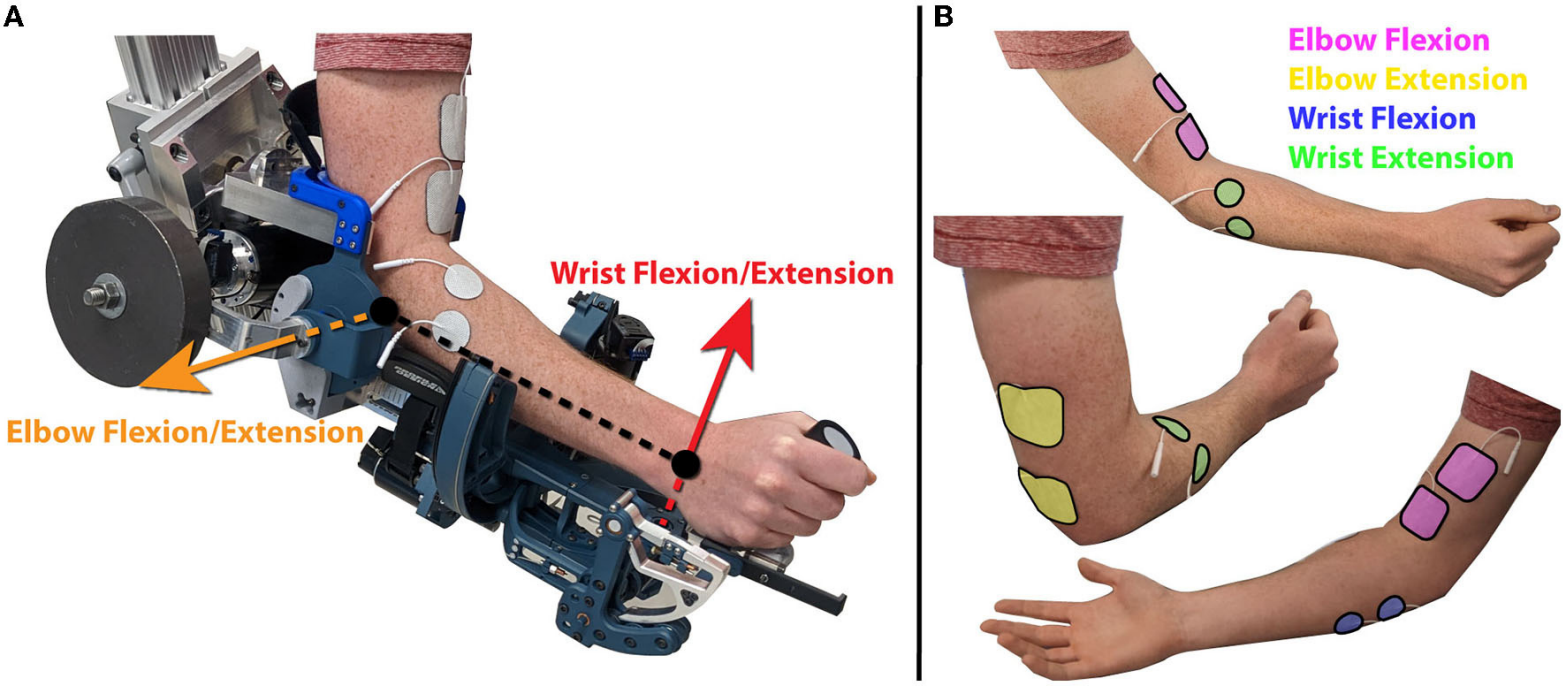
**(A)** A participant with their arm in the robot in the experimental setup, with the axes of rotation for the active joints indicated by orange and red arrows for the elbow flexion/extension and wrist flexion/extension joints respectively. **(B)** Placement of each of the four sets of electrodes. Electrodes were placed over the biceps for elbow flexion over the triceps for elbow extension. Electrodes were placed on the flexor carpi ulnaris for wrist flexion, and extensor carpi radialis longis and extensor carpi ulnaris muscles for wrist extension.

The experimental protocol began by placing the electrodes on the participants. Each of the electrode pairs were placed and tested one at a time. A set of electrodes was placed as agonist/antagonist pairs for each of the active degrees of freedom. This means for the elbow flexion/extension joint, one set of electrodes was placed to target elbow flexion, and another set was placed to target elbow extension using two inch square electrodes. For the wrist flexion/extension joint, one set of electrodes was placed to target wrist flexion, and another set of electrodes was placed to target wrist extension using one inch round electrodes. Electrode placement locations for each of these movements were chosen based on pilot testing based on which locations could reliably provide the desired movement. These general chosen locations are shown in Figure 2.

For the elbow flexion electrode placement, a reference electrode was placed, and a Compex motor point pen was used to find a specific point that generates biceps contraction, and the second electrode was placed there. For the remaining electrodes, the pair of electrodes were placed in a nominal location, and the pulse width was increased slowly. The resulting movement with the participant's arm on a table was observed, and the electrodes were adjusted if the desired movement was not produced. The electrodes were then wrapped with medical bandage to ensure that the electrodes stayed in the original location.

#### 2.4.2. Threshold identification

Once the electrodes have been placed, the minimum and maximum pulsewidth values that will be used for each participant need to be identified. The robot and arm were moved to a neutral configuration, and held there using independent PD controllers on all joints. For each electrode placed, the minimum value that produced a change in torque output in the PD controller is considered the minimum pulse width value, *pw*_*min*_. The discomfort threshold is then found by increasing the pulsewidth until the participant verbally indicates their maximum value which is still comfortable. The maximum pulsewidth value used throughout the experiment, *pw*_*max*_, is taken as a slight reduction from the discomfort threshold. A ramp from the *pw*_*min*_ to *pw*_*max*_ is then used to verify that the participant remains comfortable throughout the range, and that the *pw*_*min*_ is just below the threshold of providing torque output.

#### 2.4.3. Recruitment curve characterization

**Figure 3:**
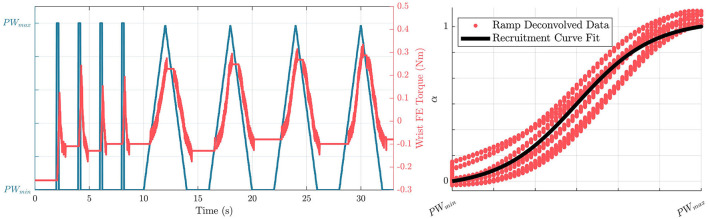
**(Left)** Profiles of commanded pulsewidths, and resulting torque outputs due to stimulation from the wrist extension electrode in the recruitment curve characterization process. **(Right)** Resulting characterized recruitment curve in the form of a sigmoid based on the ramp deconvolved data.

With the thresholds defined, a mathematical representation between the pulsewidth range and muscle activation is found, defined as a recruitment curve. Previous research has shown that functional electrical stimulation produces a muscle recruitment curve in the form of a sigmoid (Durfee and MacLean, 1989). To characterize this recruitment curve, the robot is again moved to a neutral configuration, and held there using independent PD controllers on each joint. Each of the electrodes sequentially performs four impulses at *pw*_*max*_, followed by four linear ramps between *pw*_*min*_ and *pw*_*max*_, as shown in Figure 3.

The ramp deconvolution method is used (Durfee and MacLean, 1989) with the input of pulsewidth values and the corresponding torques generated from the stimulation to generate smooth curves to be characterized. The sigmoid is then fitted using Equation 3 with free parameters of *c*_1_ and *c*_2_, where pw^*^ and $pw_{max}^{*}$ are defined as pulsewidths normalized so that a pw^*^ value of 0, corresponds a *pw*_*min*_ as defined in Equations 1, 2.


(1) $$\begin{array}{l} pw^{*}=pw-pw_{min} \end{array}$$

(2) $$\begin{array}{l} pw_{max}^{*}=pw_{max}-pw_{min} \end{array}$$

(3) $$\begin{array}{l} \alpha^{*}=\frac{c_{1}}{1+e^{-c_{2}\left(pw^{*}-\frac{pw_{max}^{*}}{2}\right)}}-\frac{c_{1}}{1+e^{\frac{c_{2}pw_{max}}{2}}} \end{array}$$

(4) $$\begin{array}{l} \alpha =\frac{\alpha^{*}}{c_{1}} \end{array}$$

This equation results in a sigmoid with a minimum value of 0 and a maximum value of *c*_1_. The term *c*_2_ is related to the slope of the function as it crosses the midpoint. To turn this characterization into the standard definition of a recruitment curve which varies from 0 to 1, α^*^ is divided by *c*_1_ to arrive at an equation for activation, α.

#### 2.4.4. Gaussian process regression model creation

The last component needed to mathematically represent the FES subsystem is a representation of the torque output based on the arm joint configuration of the participant. A Gaussian Process Regression (GPR) model is used to characterize this relationship torque when each of the FES electrode pairs is at a maximum activation as a function of the arm configuration. In this case, the black-box representation of the GPR models also implicitly capture some of the complex muscle dynamics. For each of the degrees of freedom, eight evenly spaced positions are taken between the minimum and maximum values that each joint will see throughout the experiment. At each of these positions, PD controllers on each of the individual robot DOFs are used to keep the robot at the desired position. The exoskeleton torque required to hold the pose when no muscles are stimulated is recorded as τ_*passive*_. One electrode is increased to its maximum activation, and the exoskeleton torque required to hold that pose is recorded as τ_*hold*_. We consider the difference between the two values as the torque produced by the electrode τ_*record*_.


(5) $$\begin{array}{l} \tau_{record}=\tau_{hold}-\tau_{passive} \end{array}$$

**Figure 4:**
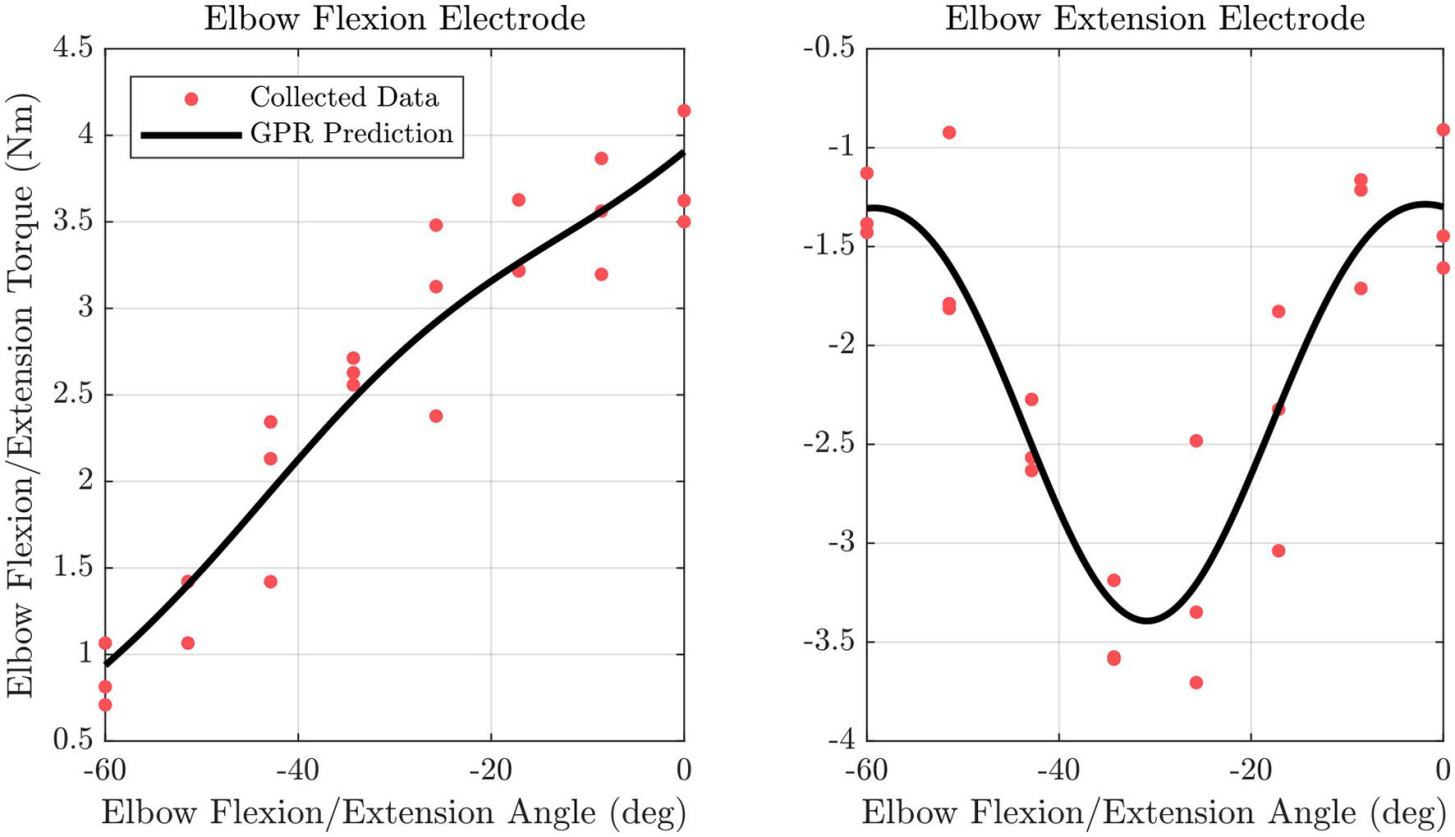
Fitted GPR models are shown along with data points used to fit the model for the elbow flexion/extension joint for the elbow flexion and elbow extension electrode.

The position tested and τ_*record*_ at that position are saved as training data for the tested electrode. This is repeated for the other electrode active for the current DOF, and at each of the other positions, three times in a randomized order. The collected training points are then used to generate a GPR model for each electrode using Matlab's fitrgp function. An example of trained GPR models for elbow flexion/extension torque output resulting from the elbow flexion and elbow extension electrodes for a single subject is shown in Figure 4. This results in the following equation


(6) $$\begin{array}{l} \tau_{fes}=P\left(q\right)\alpha \end{array}$$

where *P*(*q*) ∈ ℝ^1×2^ and where column *i* is an individual GPR model that provides an estimated output torque when electrode set *i* is at maximum stimulation, and the robot is at position *q*. Recall that this is implemented for each joint separately, so there is one *P*(*q*) that corresponds to the elbow flexion/extension joint and uses the elbow flexion/extension position as an input, and one *P*(*q*) that corresponds to the wrist flexion/extension joint and used the wrist flexion/extension position as an input.

#### 2.4.5. Arm model characterization

An accurate model of the dynamic system is needed for effective MPC implementation. Previous work has developed a model of the exoskeleton without an arm (Dunkelberger et al., 2022a). In this study, an optimization problem was solved to find an estimate of dynamic properties for the arm to be used with the exoskeleton dynamic model, including masses, moments of inertia, and friction components.

To add theese dynamic properties of the arm to the dynamic model of the exoskeleton, each joint in the arm was assumed to be a rigid body rigidly connected to the corresponding joint on the exoskeleton. With this assumption, the mass of each arm joint can be added to the mass of the robot joint, and the inertia of each arm joint can be combined with the inertia of each robot joint using the parallel axis theorem. While this study mainly focuses on the impact on the elbow flexion/extension and wrist flexion/extension joints, this arm characterization process utilizes all four joints of the exoskeleton to create a full dynamic model as shown in Equation 7, which can then be reduced to the single-joint components for the controller.


(7) $$\begin{array}{l} \tau =M\left(\text{q}\right)\ddot{\text{q}}+\text{V}\left(\text{q},\dot{\text{q}}\right)+\text{G}\left(\text{q}\right)+{\text{F}}_{\text{f}}\left(\dot{\text{q}}\right) \end{array}$$

In Equation 7, τ ∈ ℝ^4×1^ is a vector consisting of the torques at each joint. *M* ∈ ℝ^4×4^ is known as the mass matrix and consists of different combinations of the mass and inertial terms of each joint. **V** ∈ ℝ^4×1^ is the vector of centrifugal and Coriolis terms. **G** ∈ ℝ^4×1^ is the gravity vector and gives the affects of gravity on each joint, and ${\text{F}}_{\text{f}}\in {\mathbb{R}}^{4\times 1}$ gives friction on each joint. **q** is a vector of all joint positions, $\dot{\text{q}}$ is a vector of all joint velocities, and $\ddot{\text{q}}$ is a vector of all joint accelerations. *M*, **V**, **G**, and **F**_**f**_ were calculated using the same methods as previous work (Dunkelberger et al., 2022a), but with the combined arm and robot properties serving as lumped parameters in the formulation.

Equation 7 can be used to characterize the unknown arm mass properties that appear in the equation, given experimentally recorded values for τ, **q**, and $\dot{\text{q}}$. To collect these data for characterization, the user's arm was placed inside the robot and secured. A chirp signal was used as a position reference for the wrist radial/ulnar deviation joint while the other three joints were commanded to remain stationary using independent PD controllers. This process was then repeated for each more proximal joint. The torque required to complete the motions and the resulting joint positions and velocities were recorded. The recorded velocities were filtered, and a finite difference derivative was calculated to approximate the accelerations. With these values, the difference between the left side and right side of Equation 7, recorded and calculated torques respectively, could be found given a guess of mass properties. The difference between these two values at every time step is the error in the dynamic model, and this error was used as the optimization criteria to estimate the mass properties of the arm when combined with the mass properties of the exoskeleton found in previous work (Dunkelberger et al., 2022a).

To keep the number of optimization variables small, the problem was solved one joint at a time, starting with the most distal joint, wrist radial/ulnar deviation. This joint was the first to be optimized because for any given joint, only the more distal joints impact the current mass property analysis. Each more proximal joint was then optimized in order, ending with the elbow flexion/extension joint. At each joint, the inertia about the axis of rotation and the distances to the center of mass in the other two axes were optimization variables. When running the optimization on any joint except wrist radial/ulnar deviation, the next distal joint's distance to the center of mass along the distal joint's axis of rotation was also included as an optimization variable. This was added because this value does not appear in the calculations for the joint moving, but does impact the more proximal joints. Lastly, two optimization variables were added to each joint corresponding to the joint kinetic and viscous friction, which were considered to be added to the coefficients previously characterized for the exoskeleton by itself. A constant mass was assumed for each joint because the mass only appears multiplied by the distance to the center of mass terms. The formulation of this optimization problem can be seen in Equations 8, 9.


(8) $$\begin{array}{l} \underset{{\text{p}}_{\text{d}}}{\text{arg min }}e_{d}=\overset{M}{\underset{t=1}{\sum }}{\left(\tau_{calc\text{\_}d\text{\_}t}-\tau_{meas\text{\_}d\text{\_}t}\right)}^{2} \end{array}$$

(9) $$\begin{array}{l} {\text{p}}_{\text{d}}=\{\begin{array}{ll} \left[I_{czz\text{\_}d},r_{cx\text{\_}d},r_{cy\text{\_}d},r_{cx\text{\_}d+1},F_{k\text{\_}d},B_{d}\right]\; & \text{if }1\leq d\leq 3\\ \left[I_{czz\text{\_}d},r_{cx\text{\_}d},r_{cy\text{\_}d},F_{k\text{\_}d},B_{d},\right]\; & \text{if }d=4 \end{array} \end{array}$$

In these equations, **p**_**d**_ represents the vector of parameters for a given joint, *d*. A *d* of 1 represents the elbow flexion/extension joint and *d* = 4 being the wrist radial ulnar/deviation joint, *e*_*d*_ refers to the torque error between the calculated torque, τ_*calc*_, and measured torque, τ_*meas*_, *t* represents a given time step up to *M* total time steps, *I*_*czz*_ is the moment of inertia about the axis of rotation taken about the center of mass, and *r*_*cx*_, *r*_*cy*_, and *r*_*cz*_ represent the distance from the axis of rotation to the center of mass in the *x*, *y*, and *z* directions respectively.

The optimization problem was solved using fmincon in Matlab, with initial guesses of zero for all optimization variables. The optimal properties found using this method were combined into the lumped arm and robot system used in the remainder of this study.

### 2.5. Hybrid controller design

We first present the full four-DOF dynamics for the FES-exoskeleton hybrid system, which we will then reduce to the single-DOF dynamics for the control formulation. This is similar to the dynamics of the robot and arm system in Equation 7, but the inputs to the system arise from both the exoskeleton and the FES system, so we separate the torque term into the two components.


(10) $$\begin{array}{l} \tau_{\text{fes}}+\tau_{\text{exo}}=M\left(\text{q}\right)\ddot{\text{q}}+\text{V}\left(\text{q},\dot{\text{q}}\right)+\text{G}\left(\text{q}\right)+{\text{F}}_{\text{f}}\left(\dot{\text{q}}\right) \end{array}$$

In this equation, $\tau_{\text{exo}}\in {\mathbb{R}}^{4\times 1}$ and $\tau_{\text{fes}}\in {\mathbb{R}}^{4\times 1}$ are torques supplied along each of the robot joints due to robot torque outputs, and torques provided by FES respectively.

As in the previous sections, the control problem will be described once, but the equations presented apply to either the elbow flexion/extension or the wrist flexion/extension DOF. To limit the full dynamics in Equation 10 to analyze a single DOF with the rest of the joints remaining stationary, all inactive joints can be constrained such that *q*_*j*_ = *q*_*hold*_*j*_, ${\dot{q}}_{j}=0$, ${\ddot{q}}_{j}=0$ for all joints *j* that are inactive. Here, *q*_*hold*_*j*_ is the holding position of joint *j* when it is inactive, as shown in Table 1. This results with the following equation to describe the dynamics of a single DOF system, either in the elbow flexion/extension or wrist flexion/extension case.


(11) $$\begin{array}{l} P\left(q\right)\alpha +\tau_{exo\text{\_}mpc}=m\ddot{q}+g\text{ sin}\left(q-q_{eq}\right)+f_{f}\left(\dot{q}\right) \end{array}$$

For the DOF of interest, *m* represents the estimated lumped inertia, *g* represents the gravitational effects, *f*_*f*_ represents the friction effects, and *q*_*eq*_ represents the natural resting position of the combined arm-robot system for the DOF of interest. In Equation 11, and throughout the remainder of the paper, all variables that appear in equations are referring to a single DOF, and the values of these variables are different in the elbow flexion/extension DOF and the wrist flexion/extension DOF, but the symbolic expressions apply to both DOFs. For example, when this equation is applied to the elbow flexion/extension joint, *q*, $\dot{q}$, and $\ddot{q}$ are the position, velocity, and acceleration of the robot elbow flexion/extension joint, and **α** is the vector ${\left[\alpha_{1},\alpha_{2}\right]}^{T}$, which are the activation levels of the electrodes placed to induce elbow flexion, and elbow extension.

**Table 1:** Holding position of inactive joints throughout testing.

**Active joint**	***q*_*hold*_1_**	***q*_*hold*_2_**	***q*_*hold*_3_**	***q*_*hold*_4_**
Elbow F/E	N/A	0°	0°	0°
Wrist F/E	–30°	–30°	N/A	0°

To develop our control problem, we define the following quantities as the system state, *x*, system output, *y*, and and control input, *u*, where *C* is the output matrix describing the variables we can observe.


(12) $$\begin{array}{l} \text{x}={\left[q,\dot{q}\right]}^{T} \end{array}$$

(13) $$\begin{array}{l} C=I_{2} \end{array}$$

(14) $$\begin{array}{l} \text{y}=C\text{x} \end{array}$$

(15) $$\begin{array}{l} \text{u}={\left[\tau_{exo\text{\_}mpc},\alpha_{1},\alpha_{2}\right]}^{T} \end{array}$$

To use standard analysis techniques, we would like to have our dynamics in the form of $\dot{\text{x}}=f\left(\text{x},\text{ u}\right)$, which by definition is the vector ${\left[\dot{q},\ddot{q}\right]}^{T}$. By solving Equation 11 for $\ddot{q}$ as follows, we can obtain an explicit definition for the representation of *f*(**x**, **u**).


(16) $$\begin{array}{l} \ddot{q}=\frac{1}{m}\left(P\left(q\right)\alpha +\tau_{exo\text{\_}mpc}-g\text{ sin}\left(q-q_{eq}\right)-f_{f}\left(\dot{q}\right)\right) \end{array}$$

To implement real time control, it is beneficial to use a linearized form of the dynamics to reduce computation time. We can then convert the dynamics to a linearized form by calculating the Jacobian of the dynamics about time *k* with respect to the input and output. The following gives a estimate for the dynamic equations at time *i*, linearized at time *k*.


(17) $$\begin{array}{l} A_{k}={\frac{\partial f}{\partial \text{x}}|}_{\text{x}={\text{x}}_{k},\text{u}={\text{u}}_{k}} \end{array}$$

(18) $$\begin{array}{l} B_{k}={\frac{\partial f}{\partial \text{u}}|}_{\text{x}={\text{x}}_{k},\text{u}={\text{u}}_{k}} \end{array}$$

(19) $$\begin{array}{l} {\dot{\bar{\text{x}}}}_{i}=A_{k}{\text{x}}_{i}+B_{k}{\text{u}}_{\text{i}}+\dot{\text{x}}{|}_{\text{x}={\text{x}}_{k},\text{u}={\text{u}}_{k}} \end{array}$$

These linearized dynamics are then used in the MPC formulation. The cost function is as follows, where *i* represents a discrete point in time in the standard MPC formulation.


(20) $$\begin{array}{l} J_{i}={\left({\text{r}}_{i}-{\bar{\text{y}}}_{i}\right)}^{T}Q\left({\text{r}}_{i}-{\bar{\text{y}}}_{i}\right)+\Delta {\text{u}}_{i}^{T}R\Delta {\text{u}}_{i}+{\text{u}}_{i}^{T}R_{m}{\text{u}}_{i} \end{array}$$

The matrices *Q* ∈ ℝ^2×2^, *R* ∈ ℝ^3×3^, and $R_{m}\in {\mathbb{R}}^{3\times 3}$ are positive diagonal matrices used to weight predicted trajectory error, control input rate of change, and control input magnitude respectively. In this equation, the control input rate of change at timestep *i* is defined as Δ*u*_*i*_ = *u*_*i*_−*u*_*i*−1_. Initial values for these gains were chosen based on pilot studies that provided desired behavior as described below.


(21) $$\begin{array}{l} Q=\left[\begin{array}{cc} Q_{pos} & 0\\ 0 & Q_{vel} \end{array}\right] \end{array}$$

(22) $$\begin{array}{l} R=\left[\begin{array}{ccc} R_{exo} & 0 & 0\\ 0 & R_{fes} & 0\\ 0 & 0 & R_{fes} \end{array}\right] \end{array}$$

(23) $$\begin{array}{l} R_{m}=\left[\begin{array}{ccc} R_{m\text{\_}exo} & 0 & 0\\ 0 & R_{m\text{\_}fes} & 0\\ 0 & 0 & R_{m\text{\_}fes} \end{array}\right] \end{array}$$

The general ideology behind the choice of gains in the hybrid controller is as follows. The gains for *Q* represent the importance for the controller to follow the desired trajectory, with higher gains indicating better tracking, but less stable behavior if there are model errors. The gains for *R*_*m*_ are chosen so that *R*_*m*_*exo*_ ≫ *R*_*m*_*fes*_, which is the main method by which the hybrid control strategy reduces exoskeleton torque compared to a strategy which only uses an exoskeleton. Additionally, these gains are chosen such that ${\left({\bar{\text{y}}}_{i}-{\text{r}}_{i}\right)}^{T}Q\left({\bar{\text{y}}}_{i}-{\text{r}}_{i}\right)\gg {\text{u}}_{i}^{T}R_{m}{\text{u}}_{i}$, so that trajectory accuracy is not sacrificed to allow for overall torque reduction. The gains for *R* are chosen so that *R*_*fes*_ ≫ *R*_*exo*_ so that the FES system, which has significant delay, remains stable by mainly responding with low-frequency changes in torque while the exoskeleton does mostly quick corrective actions. This combination of chosen gains for *R* and *R*_*m*_ are intended to have the general effect of the FES subsystem providing low frequency, high amplitude torque, allowing it to provide a bulk of the power requirement, yet maintain smooth motions despite the time delay. The exoskeleton subsystem provides high frequency, low amplitude torque, which provides necessary quick corrections without requiring too much power consumption. As a reminder, separate controllers are used for the elbow flexion/extension joint and for the wrist flexion/extension joint, and the gains for each of the two joints are created independently.

Because the *R*_*fes*_ and *R*_*m*_*fes*_ gains place costs on activation levels rather than FES torque outputs, in some cases, it was necessary to adjust these values for each participant upon initial testing with the hybrid controller to account for variations in torque productions for the same activation level. To account for this, when the hybrid controller was first tested in the experiment, these gains were increased by a factor of two from the original values if there was oscillatory behavior, or decreased by a factor of two if activation levels were lower than expected.

The final cost function used in the MPC implementation is as follows.


(24) $$\begin{array}{l} \underset{u\left(\cdot \right)}{\text{arg min }}J_{tot}=\overset{N}{\underset{i=1}{\sum }}J_{k+i}\\ \text{subject to }{\bar{\text{y}}}_{k+i+1}={\bar{\text{y}}}_{k+i}+{\dot{\bar{\text{x}}}}_{k+i}T_{s},\\ 0\leq \alpha_{e}\leq 1,e=\left\{1,2\right\} \end{array}$$

In Equation 24, *k* represents the current point in time, and future discretized timesteps at time *k* + *i* are *T*_*s*_ seconds apart, for *N* time steps. The dynamics at these future time points are approximated using Euler integration as shown by the fist constraint on the optimization problem, with the bars representing that these are estimated values. The second constraint restricts the activation level, α, of each electrode, *e*, to fall between 0 and 1. An additional constraint could be implemented to limit the maximum allowable exoskeleton torque; however, in this study, the torque required from the exoskeleton always remained below the maximum allowable torque, which meant that this constraint did not need to be implemented. The result of the optimization is *u*(·) which represents the optimal control inputs over the time prediction horizon, *u*_*k*+1_, *u*_*k*+2_, ..., *u*_*k*+*N*_.

This MPC formulation is created in C++ using the nonlinear optimization framework CasADi (Andersson et al., 2019). The solver for the dynamic problem is compiled into a dll file which can be loaded at runtime and interfaced with the Interior Point Solver, IPOPT (Wächter and Biegler, 2006), to solve the MPC problem. This MPC problem is solved as fast as possible in a separate thread, and each time a solution is found, the solution of the minimization, *u*(·), is sent to the main thread, where those successive control solutions are used until the next solution is found. From *u*, τ_*exo*_*mpc*_ is used directly, and α_1_ and α_2_ are converted to pulsewidth commands to send to the stimulator using Equation 4 which describes the recruitment curve.

To tune the gains for the MPC algorithm, *Q* and *R* were first tuned to achieve smooth movements and low tracking error, with *R*_*m*_ values kept at 0. Following this, the *R*_*m*_ gains were chosen to achieve meaningful reduction in the exoskeleton torque, while maintaining similar tracking accuracy. As *R*_*m*_ gains were tuned, *Q* and *R* were further adjusted as necessary.

**Figure 5:**
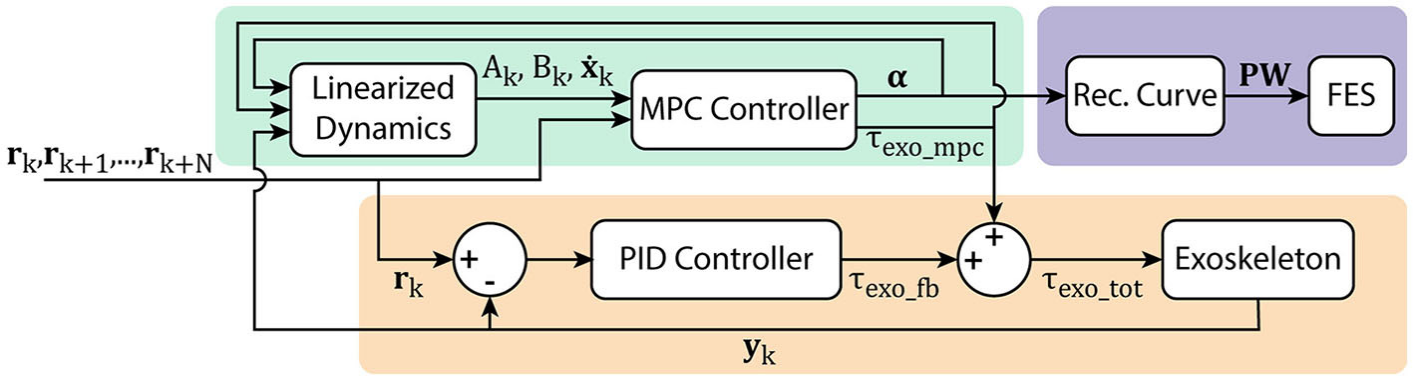
Hybrid FES-exoskeleton control block diagram, showing how the different components of the hybrid controller work together to provide torque commands to the robot and pulse width commands to the stimulator given a desired input trajectory.

To account for model error in the MPC formulation, a PID controller using only exoskeleton torque is implemented in parallel as shown in Figure 5. This has the effect of allowing the MPC portion to control most of the action, while still providing a high accuracy on the resulting trajectory tracking. The torque provided by the PID controller is defined as τ_*exo*_*fb*_, and the gains for this controller were chosen in pilot testing to achieve between 1 and 1.5° RMS tracking error. In the tuning of this controller, the gains were slowly increased, and tuned only after fully tuning the MPC system independently, so that the controller dynamics achieved from the MPC algorithm were the driving component. This additional controller does not change the output applied by the FES subsystem, but the torque applied to the exoskeleton becomes


(25) $$\begin{array}{l} \tau_{exo\text{\_}tot}=\tau_{exo\text{\_}mpc}+\tau_{exo\text{\_}fb} \end{array}$$

To test the effectiveness of the hybrid controller design, it is compared against a purely exoskeleton controller, defined as the *exoskeleton alone* control case. In this test case, the same general structure is used with the MPC controller paired with a PID controller, but Equation 15 becomes


(26) $$\begin{array}{l} \text{u}=\left[\tau_{exo}\right] \end{array}$$

which results in *R* and *R*_*m*_ being single values rather than matrices.

### 2.6. Experimental validation

After a participant completed each of the model characterization steps and the MPC problem was generated, the experimental validation was conducted. Participants were assisted in completing two different trajectories in two different conditions, using the hybrid controller that combined the FES and exoskeleton action, and using the exoskeleton alone. The first trajectory is referred to as the *cup* trajectory, and it is based on a study that tracked healthy individuals' joint-level movements to move a cup to various target locations with differing grasps (Valevicius et al., 2019). The movement profile for each of the joints was taken independently and spaced so that it spanned a useful and comfortable trajectory space for the exoskeleton used in this study which was 30° flexed to 90° flexed from full extension for elbow flexion/extension and 15° extended to 45° flexed for wrist flexion/extension. The *cup* trajectory is useful to observe how the hybrid controller behaves when following natural motions that would be expected under normal use. The second trajectory is referred to as the *sinusoidal* trajectory, and it is an artificially created trajectory that is the summation of multiple sinusoidal waves at different amplitudes and frequencies. This trajectory was created to test the controllers' ability to generalize to different movements. The trajectories are relatively similar in terms of difficulty for the elbow flexion/extension joint, but the wrist flexion/extension joint movement is significantly easier in the *cup* trajectory than the *sinusoidal* trajectory. Both trajectories take 42.4 s to complete, which is four times the time it took an average able-bodied individual to complete the *cup* trajectory in Valevicius et al. (2019). A four times reduction was chosen because the original trajectory moved through the workspace very quickly, and this reduction empirically felt an appropriate length to safely perform movements with a human in the robot. Visualizations of these trajectories are shown in the results in **Figures 8**, **9**.

Each DOF was tested for ten trials on the *cup* trajectory, split evenly between hybrid controller and exoskeleton alone controller, and 10 trials on the *sinusoidal* trajectory, also split evenly between hybrid controller and exoskeleton alone controller. While each DOF was being tested, all other DOFs were kept at their *q*_*hold*_ values as shown in Table 1 using independent PD controllers on those joints. Collection of the experimental data began by running four elbow flexion/extension trials, consisting of one of each possible combination of trajectory and controller type. This was followed by four wrist flexion/extension trials, again consisting of each possible combination of trajectory and controller. This sequence was repeated until all 40 total trials had been collected. Throughout each of the trials, position of the active DOF, total exoskeleton torque commanded, and activation levels of electrodes were collected at a rate of 1 kHz using a Quanser Q8-USB data acquisition device.

Three of the nine participants repeated the entire protocol (including characterization steps) at least 1 week after they completed the first set of data collection. These data were collected to provide insight into whether results remain similar between sessions within the same participant, rather than only comparing between participants.

### 2.7. Data analysis

The primary objective of these experiments is to understand the extent to which exoskeleton power consumption can be reduced in a hybrid system compared to a exoskeleton alone system. We compare power consumption by taking the sum of the squared total exoskeleton torque throughout the trajectory for each of the conditions tested as shown in Equation 27, averaged across each of the five trials with that set of conditions. This value is labeled as τ_*ss*_*exo*_ for the exoskeleton alone control condition, and τ_*ss*_*hybrid*_ for the hybrid control condition. Because participants have different arm sizes, and require the robot to be in different configurations, it is expected that participants will require different amounts of sum of squared torque from the system to move through the *cup* and *sinusoidal* trajectories. To normalize the data to compare across subjects, the reduction in sum of squared torque in the hybrid control case compared to the exoskeleton alone control case is shown by Equation 28. This allows us to analyze the varying power consumption both between exoskeleton alone and hybrid controllers, as well as how the relative controller performance translates between two different trajectories.


(27) $$\begin{array}{l} \tau_{ss}=\overset{N}{\underset{i=1}{\sum }}\tau_{exo\text{\_}tot}^{2} \end{array}$$

(28) $$\begin{array}{l} \%Imp=100\left(1-\frac{\tau_{ss\text{\_}exo}-\tau_{ss\text{\_}hybrid}}{\tau_{ss\text{\_}exo}}\right) \end{array}$$

In Equation 27, *N* is the number of data points collected. With this representation, a value of *%Imp* = 0 would represent equal amounts of torque being used in both control cases, which would indicate no improvement, a value of *%Imp* > 0 would indicate a reduction in power consumption using the hybrid controller with a value of *%Imp* = 100 indicating no exoskeleton power was consumed, and a value of *%Imp* < 0 would indicate that the hybrid controller required more exoskeleton power than the exoskeleton alone case. A paired *t*-test was performed to understand whether there was a statistically significant difference between in the sum of squared torque in the exoskeleton alone control case, and in the hybrid control case for each of the trajectories.

The secondary objective of these experiments is to understand how the tracking accuracy compares when using the two options for controllers. The RMS tracking error is calculated as


(29) $$\begin{array}{l} e_{rms}=\frac{\sum_{i=1}^{N}\sqrt{{\left(y_{i}-r_{i}\right)}^{2}}}{N} \end{array}$$

A paired *t*-test was performed to understand whether there was a statistically significant difference between the RMS errors in the exoskeleton alone control case, and in the hybrid control case for each of the trajectories.

One subject was unable to get any detectable torque output from one of the electrodes on the wrist flexion/extension DOF, and therefore, did not complete data collection for that DOF. Because of this, there are nine sets of data analyzed for the elbow flexion/extension results, and eight sets of data analyzed for the wrist flexion/extension results.

## 3. Results

**Figure 6:**
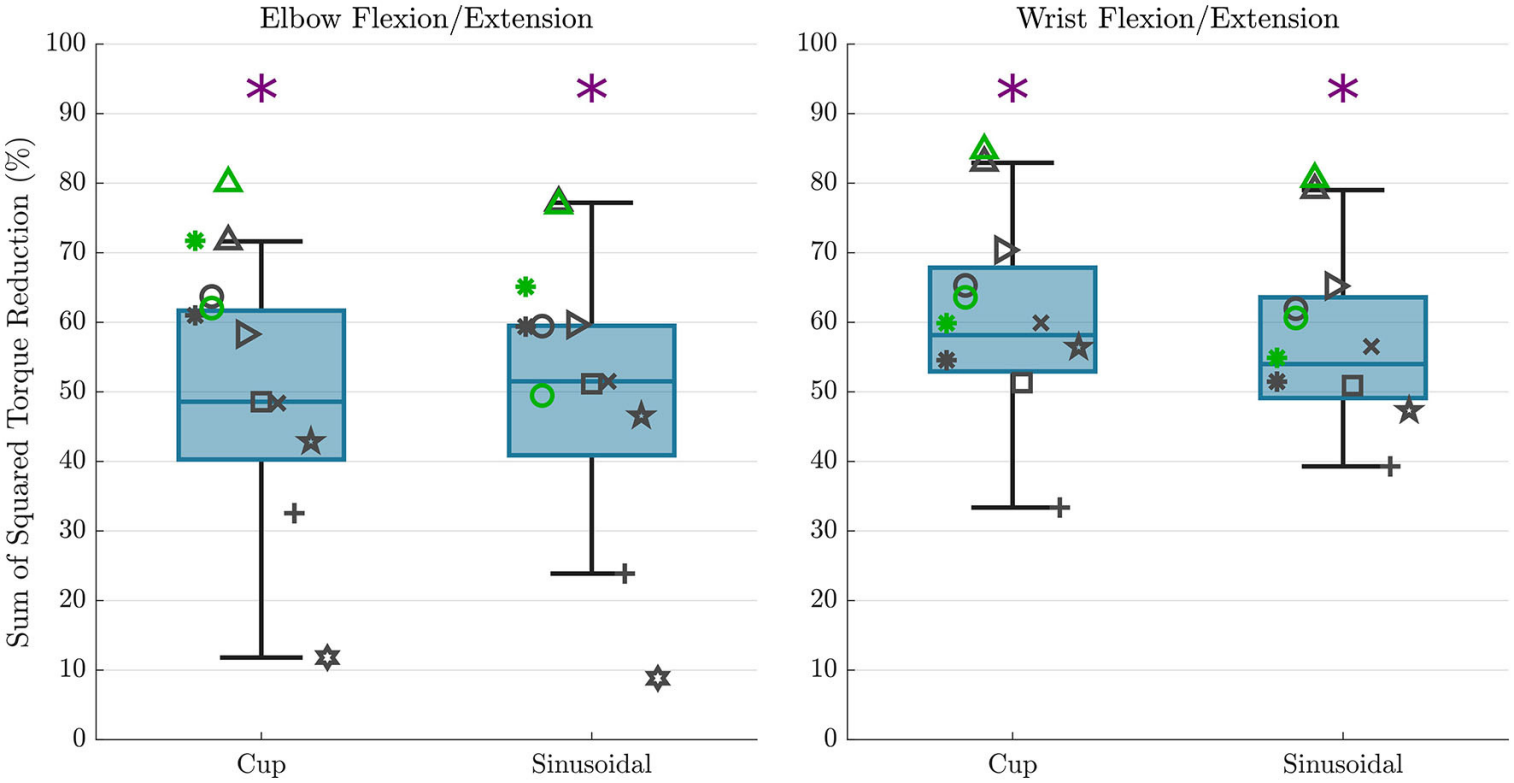
Sum of squared torque reduction results are shown for all subjects for each trajectory for the elbow flexion/extension DOF **(left)** and wrist flexion/extension DOF **(right)**. The overlaid scatterplot shows individual subject results, with the same symbol representing a single subject across figures. Points in green show the repeated data collection for the first three subjects, but repeated data collection does not contribute to boxplot presentation. The purple “*” above the plots represents a that there was a statistically significant difference in the sum of squared torque between the hybrid and exoskeleton alone control cases.

A summary of the sum of squared torque reduction findings is presented in Figure 6 as boxplots with individual subject data overlaid on top. These results show a mean sum of squared torque reduction of 48.8 and 48.6% for the *cup* and *sinusoidal* trajectories respectively for the elbow flexion/extension joint when comparing the hybrid controller to the exoskeleton alone controller. These values for individual participants spanned from 11.8 to 71.6% for the *cup* trajectory, and from 8.8 to 77.2% for the *sinusoidal* trajectory, with the lowest data point being an outlier. A mean sum of squared torque reduction of 59.3 and 56.5% was shown for the *cup* and *sinusoidal* trajectories respectively for the wrist flexion/extension joint when comparing the hybrid controller to the exoskeleton alone controller. These values for individual participants spanned from 33.4 to 82.9% for the *cup* trajectory, and from 39.3 to 79.0% for the *sinusoidal* trajectory. The statistical tests showed that the sum of squared torques were significantly lower in the hybrid control case compared to the exoskeleton alone control case in both DOFs and in both trajectories, with *p*-values being < 0.01 in both trajectories for the elbow flexion/extension joint, and *p*-values being < 0.001 in both trajectories for the wrist flexion/extension joint.

**Figure 7:**
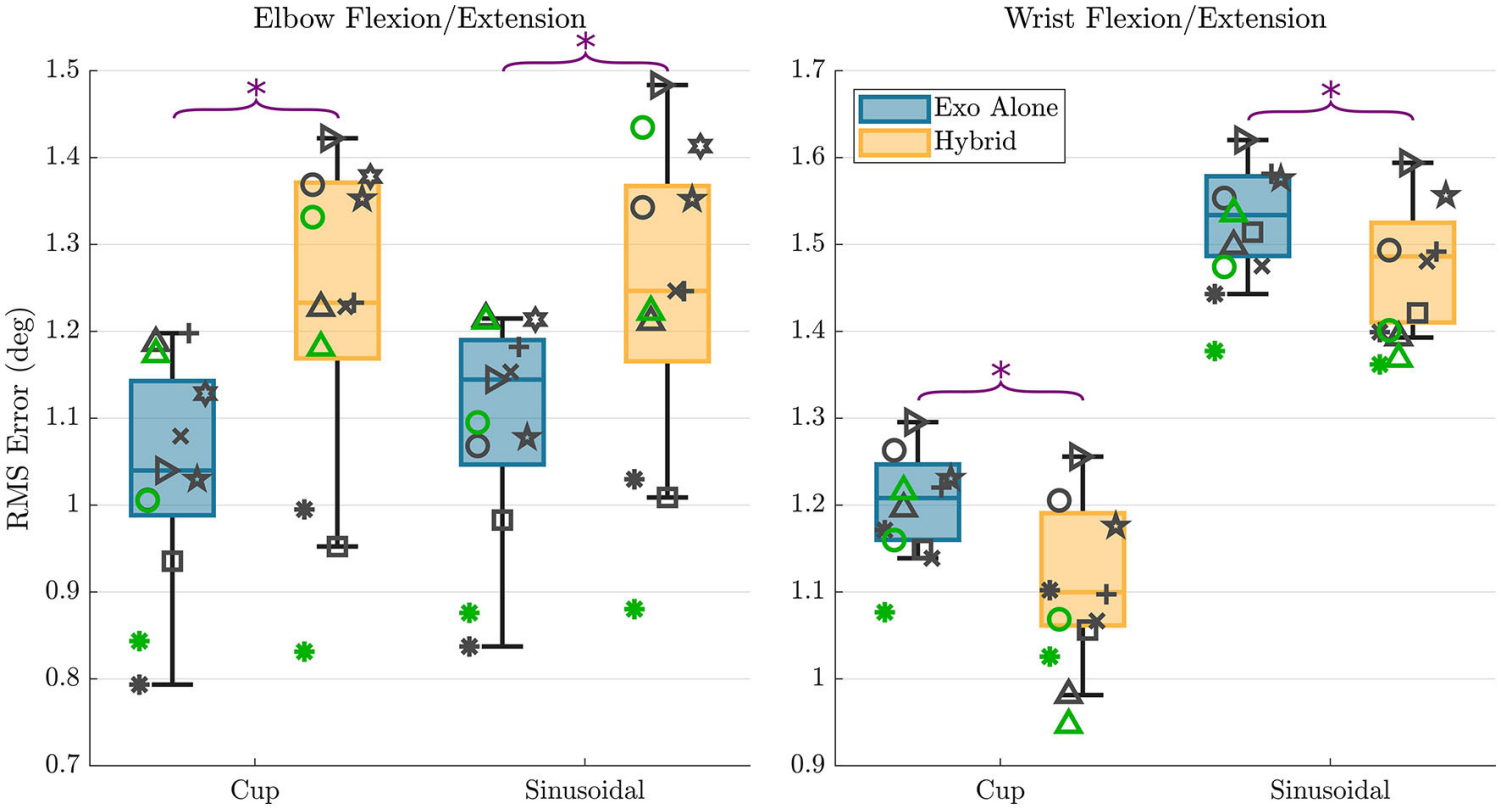
RMS error results are shown for all subjects for each trajectory and each controller type for the elbow flexion/extension DOF **(left)** and wrist flexion/extension DOF **(right)**. The overlaid scatterplot shows individual subject results, with the same symbol representing a single subject across figures. Points in green show the repeated data collection for the first three subjects. The purple “*” above the plots represents a that there was a statistically significant difference in the RMS errors between the two control types.

A summary of the trajectory tracking accuracy findings is presented in Figure 7 as box plots with individual subject data overlaid on top. For the elbow flexion/extension joint, mean RMS errors in the *cup* trajectory were 1.04 and 1.24° for the exoskeleton alone and hybrid controllers respectively. RMS errors in the *sinusoidal* trajectory were 1.10 and 1.26° for the exoskeleton alone and hybrid controllers respectively. These results indicate that there is a mean increase of 0.18° in RMS error when using the hybrid controller compared to using the exo alone controller in the elbow flexion/extension joint. This difference was shown to be statistically significant in the paired *t*-test, with *p*-values for each of the trajectories < 0.01.

For the wrist flexion/extension joint, RMS errors in the *cup* trajectory were 1.21 and 1.12° for the exoskeleton alone and hybrid controllers respectively. RMS errors in the *sinusoidal* trajectory were 1.53 and 1.48° for the exoskeleton alone and hybrid controllers respectively. These results indicate that there is a mean decrease of 0.07° in RMS error when using the hybrid controller compared to using the exoskeleton alone controller in the wrist flexion/extension joint. This difference was again shown to be statistically significant in the paired *t*-test, with *p*-values for each of the trajectories again remaining < 0.01.

**Figure 8:**
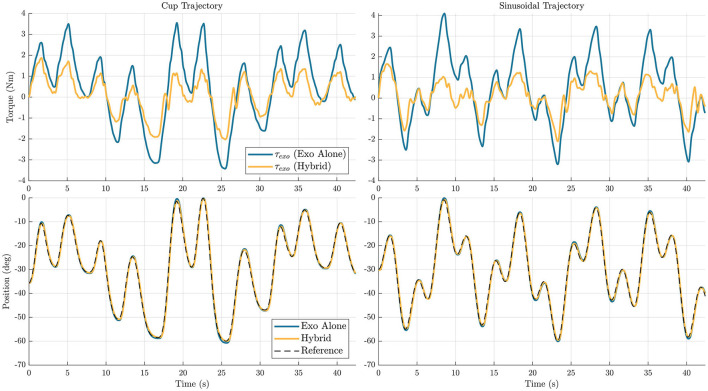
Elbow flexion/extension joint exoskeleton torque profile for a single subject **(top)**, and movement profiles averaged across subjects **(bottom)** are shown for the two different trajectories, *cup* **(left)** and *sinusoidal* **(right)**. In the plots, the blue line represents data for the exoskeleton alone controller, and the yellow line represents data for the hybrid controller.

**Figure 9:**
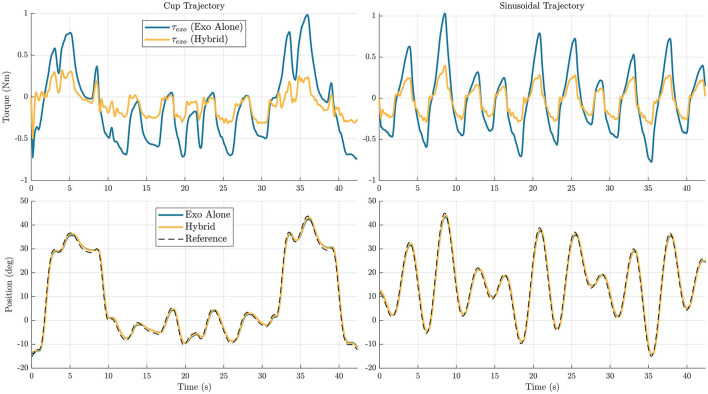
Wrist flexion/extension joint exoskeleton torque profile from a single subject **(top)**, and movement profiles averaged across subjects **(bottom)** are shown for the two different trajectories, *cup* **(left)** and *sinusoidal* **(right)**. In the plots, the blue line represents data for the exoskeleton alone controller, and the yellow line represents data for the hybrid controller.

Figures 8, 9 show time series representations of torque profiles for the best performing subject (represented by the △ symbol in Figures 6, 7) and movement profiles averaged across all subjects. In the representative plots of torque profiles, the exoskeleton torque used during the hybrid trials exhibits a smaller magnitude than the exoskeleton torque used during exoskeleton alone trials. This result shows that the hybrid controller is able to replace a significant amount of the torque requirement from the exoskeleton with FES torque. The plots for movement profiles demonstrate how well each of the controllers are able to track the trajectory. In all combinations of trajectories and DOFs, the trajectories almost entirely overlap each other, showing similar accuracy regardless of controller.

The reduction in maximum torque for the torque profile averaged across participants profiles across participants is also analyzed, for the hybrid controller compared to the exoskeleton alone controller. For this metric, it is interesting to observe both the change in maximum and minimum values, as many cable-driven systems would likely require one actuator for each agonist and antagonist pair. In the elbow flexion/extension DOF, the maximum torque was reduced by 44.2 and 43.7% in the *cup* and *sinusoidal* trajectories respectively, and the minimum torque for the mean profile was reduced by 31 and 27.1% for the *cup* and *sinusoidal* trajectories respectively. In the wrist flexion/extension DOF, the maximum torque was reduced by 67.1 and 65.3% in the *cup* and *sinusoidal* trajectories respectively, and the minimum torque for the mean profile was reduced by 36.9 and 36.6% for the *cup* and *sinusoidal* trajectories respectively.

## 4. Discussion

There is a need for devices to provide assistance in completing activities of daily living for individuals with SCI. For this population, return of upper-limb function is among their top priorities (Anderson, 2004). Both FES and exoskeletons provide some framework to assist with movement, but each of these technologies has fundamental limitations preventing meaningful assistance for the upper-limbs in activities of daily living. FES is unable to provide accurate and repeatable movements by itself, and using feedback control causes instability due to the inherent time delays in muscle response to stimulation. Exoskeletons are able to provide accurate and repeatable movements, but require bulky systems and large amounts of power to support upper-limb movements against gravity. In this paper, we have proposed a hybrid FES-exoskeleton controller that combines the two technologies, with the goal of reducing power consumption compared to a robot alone, and providing accurate movement, similar to that of an exoskeleton alone. This controller uses the model predictive control cost function to leverage the strengths of each of the subsystems, while minimizing the weaknesses of each.

### 4.1. Torque reduction

An average reduction of 48.7 and 57.9% of sum of squared torque was found on the elbow flexion/extension and wrist flexion/extension DOFs respectively with the use of the hybrid controller compared to the exoskeleton alone controller. These results in the EFE joint are an improvement over the 32.1% reduction found in our previous implementation using only the *cup* trajectory (Dunkelberger et al., 2022b). This improvement shows that the inclusion of the feedback controller instead of using an integral term, and the incorporation of a more sophisticated arm model, resulted in greater benefits in this hybrid control scheme, while even extending to more generalized trajectory cases. This shows promise for meaningful power consumption reduction for a hybrid system when comparing to an exoskeleton alone controller. Practically, this could mean that a portable hybrid system could be powered for roughly twice as long as an equivalent exoskeleton alone system, given the same battery capacity. In the future, this could lead to more portability and longevity in hybrid assistive devices for impaired populations.

It is worth noting that while the participants are able-bodied and can move their arm through the desired trajectories without assistance, we should not expect to see a torque reduction of 100%. With FES we often cannot achieve the full capabilities of the user's muscles, and in this study, many of the participants were not able to produce the maximum required torque solely through FES, even at maximum activation. Additionally, FES is known to not provide accurate or repeatable movements by itself, so at a minimum, the exoskeleton needs to provide corrective torques to account for these inaccuracies.

The average reduction in minimum and maximum torques shows potential for actuator sizes to be reduced while still achieving the same resultant motion, which would result in less bulky assistive robotic systems. In the future, this could be more directly tested by artificially limiting the maximum torque of the exoskeleton joints to observe how the FES can make up for the lack of torque.

### 4.2. Accuracy

FES systems by themselves do not provide reliable repeatability when trying to perform generalized movements. The goal of hybrid FES and exoskeleton systems is to achieve trajectory-following accuracies significantly better than FES systems by themselves, ideally approaching accuracies that are achievable using exoskeleton-alone systems. In the elbow flexion/extension joint, the hybrid algorithm had on average 0.20 and 0.16° more RMS tracking error on the *cup* and *sinusoidal* trajectories, respectively, when comparing the hybrid controller to the exoskeleton alone controller. While this was a decrease in accuracy, this still resulted in a very similar motion over the trajectory, as shown in Figure 8. To put this in perspective, for a forearm length of 30 cm, the RMS error in positioning the wrist, given the error in angular tracking, is ~1 mm. For the wrist flexion/extension joint, the hybrid controller had on average 0.09 and 0.05° less RMS tracking error on the *cup* and *sinusoidal* trajectories, respectively. Again, while there is a small decrease in accuracy, the resultant trajectories are very similar, as shown in Figure 9. These results demonstrate that the hybrid controller is able to achieve similar tracking accuracies to the exoskeleton alone controller in both of the individual DOFs.

It is worth noting the difference in tracking accuracy between the *cup* and *sinusoidal* trajectories on the wrist flexion/extension joint. Recall that the *cup* trajectory requires significantly less movement, with an average velocity of 7.3 °/s compared to the sinusoidal trajectory with an average velocity of 14.3 °/s. The difference in difficulty between the trajectories is likely the cause for more tracking error in the *sinusoidal* trajectory. Still, we see that the general relationship of the hybrid controller having a 0.06° RMSE improvement is similar to the 0.09 degree RMSE improvement on the *cup* trajectory.

A benefit of the proposed control architecture is that the feedback controller portion can be adjusted independently of the model predictive control portion. This means that if a specific movement needs high-precision, the gains of the feedback controller can be modified in a straightforward manner to increase accuracy, although it would result in an increase in exoskeleton torque usage. Additionally, while this study focused on the challenging task of tracking time-varying trajectories, it would also be an interesting translation to modify the implementation to achieve desired setpoint positions, where FES could be used for a majority of the movement generation when it is far from the target, and the exoskeleton could be used to fine-tune the position when it is close to the desired setpoint.

### 4.3. Generalization across tasks

Many of the previous applications using FES for assistance provide the stimulation using a pre-programmed profile for a specific movement. An important feature of the proposed hybrid controller is that it does not rely on knowing the desired trajectory before use, and works with any given input trajectory. By testing two different trajectories, we were able to observe how the different outcome metrics varied in different movements. Tracking performance across several tasks has been reported by a few studies that use both FES and exoskeletons (Rohm et al., 2013; Memberg et al., 2014; Ajiboye et al., 2017), but none of these studies use a controller to distribute actuation between the two systems on the same joint.

The sum of squared torque reduction was similar between the two trajectories for both the elbow flexion/extension DOF and the wrist flexion/extension DOF. Along with the means and ranges being the same, the general spacing of the participants within the range of results remained the same between the two trajectories. This means that the benefits in power reduction did generalize well to these different trajectories, and that users could expect similar results on trajectories that require similar motions. It is especially interesting that a similar level of sum of squared torque reduction was found on the two different trajectories for the wrist flexion/extension joint, especially because one of the trajectories was significantly more challenging than the other.

While the elbow flexion/extension DOF saw similar tracking accuracies in the two different trajectories when comparing the two controllers, the wrist flexion/extension DOF did see a difference in trajectory tracking accuracy on the two different trajectories. Despite this, the relationship between the exoskeleton-alone tracking accuracy and the hybrid tracking accuracy remained similar in all cases, with the elbow flexion/extension DOF showing average increase of 19.2 and 14.5% in RMS error on the *cup* and *sinusoidal* trajectories respectively, and the wrist flexion/extension DOF showing average reduction of 7.4 and 3.3% on the *cup* and *sinusoidal* trajectories respectively.

While not implemented in this paper, another benefit of this proposed controller is the ability to intuitively adjust controller behavior to generalize to different objectives of movement. If a specific task requires high precision in a movement, the gains of the *Q* matrix or feedback could be increased to favor more accurate movement at a cost of more torque. If there is an onset of fatigue, the weights of the *R*_*m*_ matrix can be adjusted to prefer more exoskeleton torque, and allow the muscles to recover.

### 4.4. Consistency across participants

**Figure 10:**
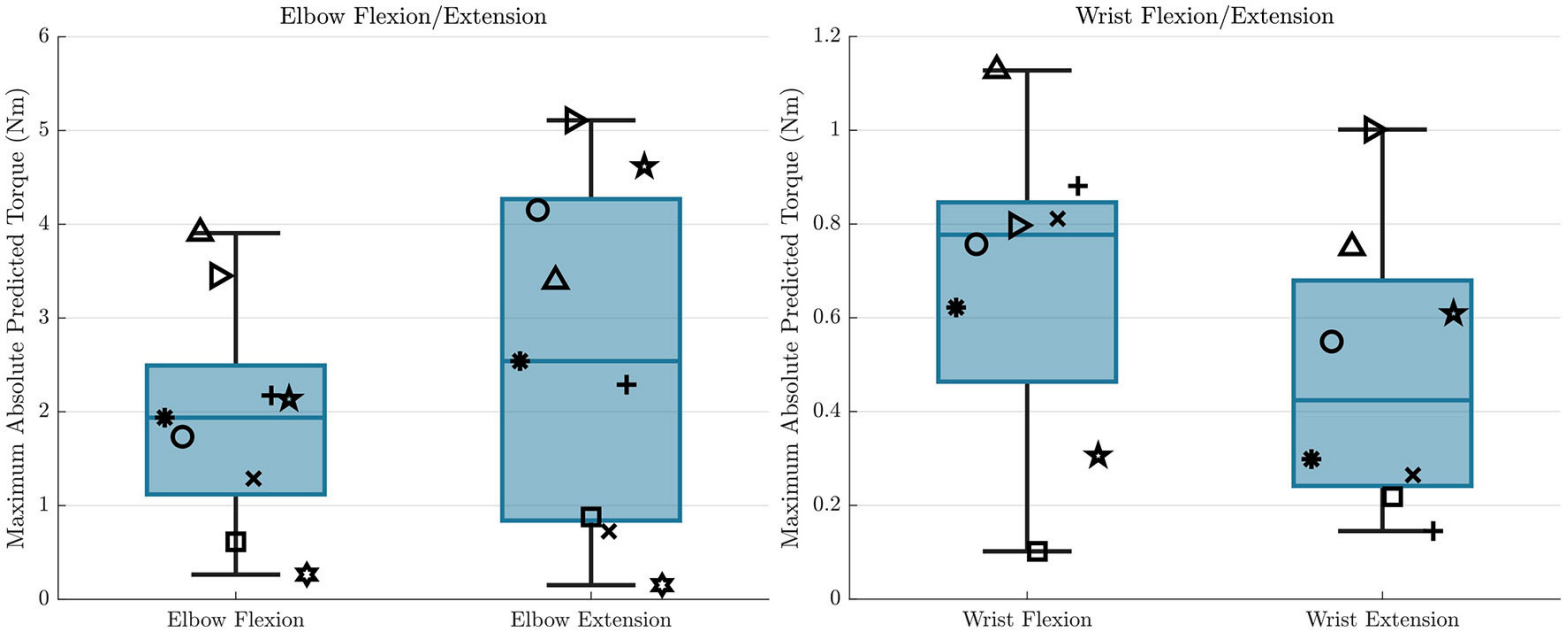
The maximum absolute values of the GPR predictions throughout the workspace for all participants are shown for each electrode, and for each DOF. This represents how different participants are able to achieve different levels of torque from FES when the participant is receiving maximum stimulation. The symbols here correspond to the same symbols from Figures 6, 7.

While the results between trajectories were consistent within participants, there is a significant distribution of results between participants, especially for the sum of squared torque reduction observed for the hybrid controller compared to the exoskeleton alone controller. Even though all results showed improvement, except for the single participant who could not achieve an FES response in one of the wrist flexion/extension electrodes, some participants had significantly better results than others. There are many factors that can impact the effectiveness of FES, including electrode placement, size of muscles, body fat levels, and fatigue, many of which are not modifiable. These variations in ability to produce torques due to FES can be visualized across participants in Figure 10, where the maximum absolute value that the GPR model predicts that each participant can produce throughout the workspace is shown. We can see that there are wide variations in the predicted amount of FES torque production. As an example, one participant cannot produce more than about 0.25 Nm of torque throughout the entire workspace with either the elbow flexion or elbow extension electrodes, but two other participants can produce more than 3 Nm in both of these cases. With these differences in mind, it is clear that some participants would never be able to achieve high reductions in power consumption with this hybrid control approach. To increase consistency between participants, it would be interesting to test with implanted FES systems, which are more reliable and targeted, and to model fatigue, which can help modify the controller in real-time to account for it.

When observing the results of the three participants who performed the same protocol twice separated by at least a week, we see that the results remained similar between the two time points. The difference between sessions in sum of squared torque reduction when comparing the hybrid controller to the exoskeleton alone controller remained within 17% across participants for the elbow flexion/extension DOF, and below 10% for the wrist flexion/extension DOF. The difference between sessions in RMS tracking error for the hybrid controller compared to the exoskeleton alone controller remained below 7% across participants for the elbow flexion/extension DOF, and below 12% on the wrist flexion/extension DOF. It is encouraging that even though it is difficult to generalize across participants, these preliminary repeatability results seem to indicate that results hold steady within users if the same implementation procedure is followed during each use. It is important to note here that the participants repeated the entire protocol, and it is expected that the model that the FES production will change (especially when using surface electrodes), meaning that the model will necessarily have to be tuned for each use, even for the same participant.

One factor of this controller implementation that does not generalize across participants is that it relies on the relative weighting between exoskeleton torque inputs and FES activation levels. While the exoskeleton torque outputs are relatively consistent across participants, the activation levels do not map directly to torque outputs, because each participant produces a different amount of torque, given an activation level. In this case, the *R*_*fes*_ and *R*_*m*_*fes*_ parameters as defined in Equations 22, 23 must be scaled for each participant, based on the torque outputs expected from the GPR models. However, once the parameters are scaled once they should only need to be modified if electrodes need to be moved, or if fatigue occurs.

One participant had a particularly weak response to the FES, with a very low response from the elbow flexion/extension electrodes, and no response from the wrist flexion/extension electrodes. This difference compared to the remainder of participants shows the importance in characterizing each individual's FES behavior to understand the potential effectiveness of using the proposed hybrid controller.

### 4.5. Future work

An area of interest in observing the behavior of hybrid systems would be to identify how maximum torque allowed by the exoskeleton changes the resulting behavior in terms of torque output and tracking error. We observed the maximum torque used by the exoskeleton in this study, but it was not limited in any particular way to influence controller behavior. We should expect the controllers to behave differently if the maximum torques are limited at the start, as the future-looking MPC controller is able to predict a torque limit onset and proactively compensate for it.

Modeling of fatigue is another area of interest when using FES, and has received much attention in the FES research community. While this study aimed to keep the stimulation time to a minimum to reduce the effects of fatigue, there were likely at least some effects of fatigue present in results. Modeling and compensating for fatigue would be a meaningful addition to the hybrid controller to see improved performance.

The overall results from this study show promise for power reduction while maintaining high accuracy when performing movements with a single-DOF through the implementation of the hybrid FES-exoskeleton controller. Importantly, these algorithms should translate to a multi-DOF use case with only small modifications. To realize truly shared control for generalized upper-limb movements, these algorithms should be tested in multi-DOF circumstances to understand potential benefits and complications in this scenario.

## 5. Conclusion

In this paper, we presented a model-based control approach to hybrid FES-exoskeleton control. We experimentally demonstrated the benefits of using this model-based controller to distribute robot and FES contributions to control elbow and wrist movements with a hybrid FES-exoskeleton system. This control strategy reduced exoskeleton torque for the hybrid system with similar tracking accuracy compared to using the exoskeleton alone. To realize practical implementation of hybrid FES-exoskeleton systems, the control strategy requires translation to multi-DoF movements, achieving more consistent improvement across participants, and balancing control to more fully leverage the muscles' capabilities.

## Data availability statement

The datasets presented in this study can be found in online repositories. The names of the repository/repositories and accession number(s) can be found at: https://github.com/mahilab/SingleDofHybridControlDataFrontiers/releases.

## Ethics statement

The studies involving human participants were reviewed and approved by Rice University IRB #FY2017-461 and Cleveland State University IRB #30213-SCH-HS. The patients/participants provided their written informed consent to participate in this study.

## Author contributions

ND, JB, ES, and MO'M contributed to conception, design of the study, and wrote sections of the manuscript. ND conducted the experiments, performed the statistical analysis, and wrote the first draft of the manuscript. All authors contributed to manuscript revision, read, and approved the submitted version.
